# Current political uses of sport revised: beyond public diplomacy and sportswashing

**DOI:** 10.3389/fspor.2024.1316732

**Published:** 2024-08-16

**Authors:** Rafael Costa, Marcelo Moriconi

**Affiliations:** Iscte - Instituto Universitário de Lisboa, Center for International Studies (CEI-Iscte), Lisbon, Portugal

**Keywords:** political uses of sport, sport diplomacy, public diplomacy, soft power, sportswashing, commercial and corporative diplomacy, sport values

## Abstract

The successful emergence of the Gulf states as central players in the global sporting ecosystem has revived the intellectual debate on the political and diplomatic use of sport. In the last decade, the amount of research on the topic have radically increase. The old narrative traditions explaining sport diplomacy have recently been joined by a new set of literature about the concept of sportswashing, which questions the place of non-democratic governments in the sporting world. Considering that current approaches are somehow limited to explain the real scope of the situation, this article proposes a new systematization of the political and diplomatic use of sport today, differentiating between practices, actors, and objectives. The aim is to demonstrate how sport as a tool of soft power has transcended the limits of public diplomacy and goes far beyond the simplistic and preconceptual analysis developed by authors who support the concept of sportswashing. The new conceptual framework warns that the politicization of sport is not always positive and shows how some political practices can jeopardies the core values of sport and delegitimize its positive aspect. The results open a new agenda for political science research on a topic that, despite its interest and importance, remains understudied.

## 1 Introduction

In recent years, the political and economic use of sport has reached controversial points, on the one side, because of the features of the governments and political leaders that use sport for their political aims and, on the other, due to the scope of practices and influence that sport governing bodies have accepted or tolerated from certain governments and political institutions from countries with little, if any, sporting traction. Countries such as Qatar, Saudi Arabia and the United Arab Emirates have launched modernization and development programs that include sport as a key sector (1–4). The implemented actions from these programs go beyond the traditional diplomatic use of sport and aim not only at organizing and hosting sporting events (5–7) - including annually hosting foreign “national tournaments”, such as the Spanish Supercopa-, but also at using national investment funds to purchase foreign flagship clubs, promoting national flag carriers through sport, recruiting sports stars to promote their countries, create institutions to lead transnational political issues (such as the creation of the International Centre for Sport Security seeking to lead the sport integrity industry), and even creating tournaments and/or sports organizations that break the traditional status quo of international sports (2, 8, 9).

This situation, mainly fueled after the election of Qatar as the host of the 2022 FIFA World Cup, has opened a debate about the objectives and values that politicians pursue using sport as a soft power tool and the role sport governing bodies play in promoting and protecting human rights, good governance, and democracy (10, 11). The literature about the diplomatic use of sport has grown in the last decade (9, 12) and the debates about sport diplomacy were complemented with a new literature around the concept of sportswashing, understood as a deliberate use of sports soft power in seeking to alter a tarnished global reputation (13).

Nevertheless, both concepts, sport diplomacy and sportswashing, seem to maintain a strong bias—and sometimes ideological charge- that limits their usefulness in correctly describing how sport is currently used by contemporary governments.

Despite the blending nature of literature on sport diplomacy, the political use of sport is generally depicted as positive, as most of the studies are based on the idea of defending sports and promoting its good impact. Most of these works understand sport as an effective means to positive ends, as many of them are based and focused on a limited group of states, predominantly from western Europe and the United States of America (14). As Postlethwaite et al. (12) demonstrated, wealthy areas of the globe such as Europe and North America represent 65% of the papers concerning the political use of sport and sports diplomacy (published between 2000 and 2020). On the contrary, regions such as Africa and South and Central America have a minimal representation. Moreover, the concept sport diplomacy has been used to describe a huge set of practices, from the use of diplomacy for sport outcomes to use sport as a political tool. Thus, it is very difficult to clearly understand what we are talking about when we talk about sport diplomacy.

In contrast, the literature on sportswashing emerged, and expanded, from analyzing cases of the political use of sport that are considered negative and that, with a few exceptions [(15), for the 2022 FIFA World Cup], have not found their way into the literature on sports diplomacy (12). In the scarce literature on the subject, sportswashing is mainly promoted by non-western authoritarian governments seeking to improve their global reputation and hide human rights violations in their countries. The concept has been used to negatively analyze political programs that include sport as an engine of economic development promoted by countries such as Russia, Saudi Arabia, or Qatar (3, 11, 16, 17). However, the ideological burden of most authors who have used this concept does not allow for an in-depth analysis of the type of actions and programs that are developed through sport, as well as the possibilities that these programs present to generate global scrutiny and promote positive social change (9).

In this framework, Postlethwaite et al. (12) claims about the lack of theoretical consistency in the literature of sport diplomacy and the need to operationalize the political use of sport in a more appropriate and useful way. It is not only a matter of systematizing in an appropriate way the actors involved and the practices they develop, but also of schematizing the drivers and means used in each type of practice. In this sense, the article argues that the concept of sport diplomacy has fallen short to describe the current political uses of sport and that it is necessary to divide between the use of sport for political purposes and the use of politics for sporting purposes. At the same time, it is necessary to differentiate such practices in the political sphere (sports diplomacy) from those that are based on economic or commercial diplomacy to pursue sporting aims or goals through sport. Based on these assumptions, and through an integrative review of the literature, the article proposes a new systematization that differentiates between different diplomatic uses of sport, different economic uses of sport, and the use of sport as a political platform. Typology is based in the means used, the objectives pursued, and the actors involved.

The new conceptual scheme warns that the politization of sport is not always positive and shows how some political practices might jeopardize sport's essential values and delegitimate its positive aspect. The results give a new approach for analyzing the political use of sport without falling in limited visions and open a new agenda for political science research on a topic that, despite its interest and importance, remains understudied.

This article is divided into three parts. The first part analyses how public diplomacy has risen opposed to the traditional state-centered diplomacy, along with the increasing importance of new non-state actors and the introduction of sports diplomacy as a form of public diplomacy. This section reviews the literature on sports diplomacy focusing on the different cases, actors and objectives that serve as the foundation for this concept and shows how the limitations of the concept of sportswashing for an in-depth analysis of contemporary political use of sport.

Then, the new framework for analysis is presented. The new systematization of current political use of sports is based on the means used, the objectives, and the actors involved.

The final part discusses the usefulness of the new analysis scheme and the political, economic, and social implications that the new framework for the political utilization of sport generates.

## 2 Public diplomacy and soft power: sport in politics and politics in sport

Sport is seen as a powerful and effective means for diplomatic and political influence on the global stage, fostering international cooperation, and achieving social goals and economic development (5, 18–23). It has been used in a political manner for a long time, and it started to emerge with the appearance and growth of the ideas of public diplomacy and soft power.

Along the XX Century, diplomacy and international relations were mainly considered an “exclusive preserve of foreign ministries” (24), and “from the state's perspective” (25). This idea, however, is outdated in the current world, as diplomacy more and more “involves many more participants who are experts in matters other than diplomacy” (26), and non-state actors have gradually placed themselves into the sphere of international relations, politics, and development policy (27).

The progress of this multi-actor international framework developed the concept of “public diplomacy”, a modern type of diplomacy characterized by not being a “uniquely stately activity, even though it stresses the practice of states” (28). It represents a collaborative and relational world (29) where other sorts of actors are involved in the management of global matters (26). States now “share the global stage with public and private entities, with whom they must also share the machinery of global politics” (26), and international representation has clearly progressed. The so-called non-state actors, that Hawks and Uzunoğlu (30) defines as “entities that participate in or act upon international relations, and (…) have sufficient power to wield influence and cause changes even though they do not belong to established state institutions”, have gained a significant importance.

This idea of using alternative forms of power resources (with a focus on persuasion) in order to achieve a goal rather than military firepower or intimidation was coined by Joseph Nye, who conceptualized it as “soft power”. Nye differentiates between hard power, the capability to coerce and induce other states to act in a particular way that contrasts with their initial will, and soft power, which he defined as an ability to obtain a state's goals through attraction (31).

While realist scholars regarded power as possessing enough material resources to be able to lead other states to perform in a certain way (32) and measured a nation's strength in the international panorama mainly by its military capacity (considered the central point of international politics) (33), Nye emphasized how resources that are not possible to be measured quantitatively play, as well, a highly significant part in the equation, getting “nations to voluntarily do what soft power nations would like them to do” (34), avoiding coercion.

Despite its important role as soft power, political scientists have not paid much attention to sport (35) and, until the past decade, the diplomatic use of sports remained an underexplored theoretical and practical area in diplomatic studies (36).

In the last decades, the term “sport diplomacy” and the notions associated with it have grown and appealed to interest around the actors involved in it, to the main goals of this branch of public diplomacy, the different cases of study, among other elements. Murray (37) defines sport diplomacy as “the conscious, strategic and ongoing use of sport, sportspeople and sporting events by state and non-state actors to advance policy, trade, development, education, image, reputation, brand and people-to-people links”.

There is an extensive literature on the political use of sport mega-events (4–7, 38). Holding sporting events is considered an effective way to achieve various objectives such as acquiring international prestige, gaining soft power, achieving political and propaganda goals, or promoting a nation's human development. Although this literature is extensive, it covers only a small part of the current political use of sport and focuses only on actions promoted and developed within the national territory.

Through an integrative literature review, Postlethwaite et al. (12) demonstrate that the literature on “sport diplomacy” was very limited until the second decade of the XXI Century. From there, scientific interest in the topic began to increase.

In fact, in the last decade, the scope of the political use of sport has changed radically, mainly due to the development programs of the Persian Gulf countries, which have bet on sport as an engine of modernization and progress (2, 9). Qatar, Saudi Arabia and the United Arab Emirates have implemented investment programs in sport that modify the status quo of sport: from organizing a World Cup in 2022 that, for the first time, had to be played in winter and altering the sport's historical calendars so that players would arrive rested and in good condition, to the point of, for example, discussing the incorporation of a team based in Dubai to participate in a European continental competition: the Basketball Euroleague^1^.

Brannagan and Grix (4) analyse the use of the organisation of mega sporting events in Qatar as a means of human capital development at the national level, primarily to promote physical activity among citizens and to imbue them with the aspirations, motivation and ambition necessary to function in a competitive global economy. In this way, according to these authors, Qatar uses sport as a means to overcome the natural “resource curse” and, in so doing, create a more sustainable economy and improve the quality of life of national citizens.

The impact of the incorporation of the Gulf countries into the global sports ecosystem continues to generate commotion and to promote various studies on the phenomenon. This is precisely where the literature review by Postlethwaite et al. (12) falls short to understand “the challenges of sport management and sport development scholars” to operationalize the political use of sport in “both research and practice”, as the authors suggest. The authors, for the sake of their subject and being a correct methodological choice, limit themselves to studying the literature on “sport diplomacy”.

According to the authors, the term sport diplomacy has been generally used to describe “interactions between nation states and territories, non-state actors and individuals across the broad spectrum of global sporting, cultural, economic and political activities” (12).

In general, these are case studies through a historical perspective, written in western countries, in which sport and politics are brought together for purposes that are presented in a positive way, or with positive outcomes, such as ping pong diplomacy (39) or the role of sport in times of Apartheid in South Africa (40, 41).

With few exceptions (15, 42), the analysis of the political use of sport by Persian Gulf countries has not been given much space in the sports diplomacy debate.

However, the situation changes radically when attention is turned to another literature that analyses the political use of sport in a negative way: the sportswashing literature. According to Boykoff (43), sportswashing is a “phenomenon in which political leaders use sport to appear important or legitimate on the world stage, while fueling nationalism and diverting attention from chronic social problems and human rights issues on the home front”. The sportswashers are wealthy autocratic states that invest in international sport as part of their public diplomacy (44), with the objective of repositioning or promoting themselves as modern, liberal, and Western-friendly, also gaining attraction through their association with world sport (45).

The concept gained popularity during the run-up to and staging of the FIFA World Cup in Qatar and promoted an incipient production of articles on the subject (3, 13, 16, 43, 46–49). In this literature, the main targets have been the Arab countries, namely Qatar, Saudi Arabia and the United Arab Emirates (1–3, 16, 46, 50).

As with the term sports diplomacy, the concept of sportswashing lacks a coherent operationalization and a definition that can be used universally (9). Some authors have questioned the concept's ideological and prejudiced charge. On the one hand, the concept only targets autocratic governments. But, as Boykoff (43) has warned, “human rights violations happen daily in Western democracies too”. However, when Western democratic countries promote similar political uses of sports, these practices are understood as sports diplomacy.

Grix et al (11). promote a broader view of the phenomenon of political and economic utilisation of sport by non-democratic governments and warn that what the sportswashing narrative tries to see as a one-way process is in fact a two-way process in which not only the sportswashers gain, but also the international sport organisations and the Western countries that receive funding. According to these authors, “without the stimulus and opportunities provided by global capitalism and the West, strategies to use sport politically and economically would not be available to non-democratic regimes.

Far from being simple face washing actions, the use of sport as a factor of development and modernization in the Gulf countries is part of strategic programs (1–4, 9) that, in general, are not analyzed by those who summarize these actions as solely sportswashing.

The governments of these countries have recognized the need to diversify their economies to become less dependent on fossil fuels and to generate sustainable income from new sectors, promote their own development and maintain a high standard of living for new generations of citizens (3, 51). Sport is one of the strategic sectors to achieve these goals (1, 2, 50–53). During an interview with Fox News, Crown Prince Bin Salman, Prime Minister of Saudi Arabia was asking about sportswashing practices and, emphasizing the success of the Saudi Arabian political use of sport, he ironically confirmed: “If sport washing is going to increase my GDP by way of 1%, then I will continue doing sport washing”^2^.

On the other hand, the result of Arab countries' success in sport has generated increased scrutiny of their domestic politics at the global level. The case of the Kafala labor system in Qatar is a crucial example: Qatar has been accused of human rights violations and enormous death rates due to this enslaving labor regime (54). Although Kafala has existed for years, the phenomenon only entered the agenda of the political use of sport because Qatar hosted the 2022 World Cup. Even worse, as Moriconi (9) points out, references to Kafala in the Western press and in the sportswashing literature refer only to Qatar, when it is also a labor regime practiced by other countries such as Oman, Lebanon, Kuwait, and Bahrain. Is Kafala the real concern or the fact that Qatar hosted the World Cup, and it increased the country's role in the global sporting ecosystem? Why is there a lack of space in Western media for the cases of other countries?

Rather than being a problem, this is a great opportunity to use sport as a tool to promote positive values (9). Firstly, because the success of (these countries') sport strategies depends exclusively on the fact that sport governance bodies and global sport actors recognize them and open them the doors to the sport ecosystem. In practice, there is a low probability of a country hosting a Formula 1 grand prix if the FIA does not incorporate that grand prix into the official calendar. Whether Cristiano Ronaldo chooses to continue his career in Saudi Arabia is ultimately up to him. The legitimization of the possibility of political use of sport is, in these cases, always external and institutional.

Secondly, because the success of these programs (far from cleaning up political pedigrees) has generated, or can generate, cultural and political changes. In the case of Qatar, the country has committed to reforming its labor system (55). On the other hand, knowing the secondary status of women in these countries, the 2022 FIFA World Cup was the first in which a woman refereed a match^3^. On Qatari soil, and with the world watching, a woman was chosen to referee a men World Cup game.

Considering these issues that demonstrate the important role sport plays in the current international political context, as well as the understanding of the ideological limitations of the concept of sportswashing and the importance of establishing better theoretical and operational approaches to the literature on sport diplomacy (including the successful case of the Persian Gulf countries), this article presents a new systematization of practices to understand what we are talking about in terms of the political use of sport.

A coherent understanding of the practices, actors, means, and ends involved in current strategies of political use of sport is crucial to promote more and better programs in this area and to analyze the existent ones in more detail (what is done, what is not done, what could be done).

Finally, and considering the criticisms received by authors who defend the idea of sportswashing, a coherent systematization of practices serves to understand how the legitimization processes of actions take place and, in this way, understanding the areas of risk that could jeopardize the core values of sport.

## 3 Current political uses of sport: a new framework for analysis

What some critics disparagingly define as sportswashing is actually an efficient multi-actor sports strategy with clear social, economic, and political goals (1, 2, 11).

The breadth of practices and actions promoted by these successful programmes transcends traditional sports diplomacy, which has been devoted to analysing the political use of mega sport events (at the national level, such as hosting the Olympic Games), the causes and consequences of investing in and promoting elite sport (at the national level), the promotion of bilateral sport events to improve international relations (as in the case of Ping Pong Diplomacy). Today, Gulf countries have gone far beyond national borders and, in some cases, have changed the status quo of international sport by acquiring foreign clubs and turning them into real powers in their sports, hosting not only international mega-events, but also national events in other countries (such as the Spanish Copa del Rey); creating and running new international professional circuits (as in the case of golf or padel).

To understand the scope and windows of opportunity that the new programmes for the political use of sport have been able to exploit, it is necessary to analysis the actors involved, the means and elements that make each of these practices possible. On the other hand, the list of actions is so diverse that it includes practices in which sport is the actor and others in which sport is the recipient, practices in which diplomacy is the central tool and others in which the possibility of success and the raison d'être is simply economic power. In this case, rather than talking about sport diplomacy, one should talk about economic or commercial diplomacy in or through sport.

In this sense, this article propose to differentiate between sport diplomacy and policy and commercial or corporate diplomacy in relation to sport. Each of these, in turn, must be understood in terms of the key actor and the means used. In this way, both diplomacies can be practiced through sport, within sport, or for sport (See Table 1). Meanwhile, there is politics related with sport where sport can be used for political outcomes and for political activism.

**Table 1:** Current political use of sport (with examples of the gulf countries).

Practice	Type	Means	Ends	Actors involved	Qatar’s examples	Saudi’s exampes	UAE’s examples
Diplomacy and politics	Through sport	Sport	Political, social, economic goals.	Politicians, Civil servants, Diplomats, ONGs, athletes	“Ping Pong diplomacy 2.0”: Peace and Sport Table Tennis Cup, 2011	“Soccer Diplomacy”, Saudi Arabia and Iran	
	Within sport	Political & diplomatic machinery	Political statement and struggles	Politicians, diplomats, sport governing bodies	Countries boycott for Qatar 2022 Football World Cup		
	For sport	Political & diplomatic resources, including public diplomacy	Sportif success, hosting sport events, run an international federation	Politicians, diplomats	Aspire Academy, Mission Passports	Record-breaking transfers of world-class players to Saudi Pro League	Hosting events on various sports: Tennis Legends Team Cup finals, Egyptian Super cup, World Paddle Tennis, Abu Dhabi Grand Prix
Commercial and corporative diplomacy	Through sport	Economic resources (money)	Publicity	Flagship companies	Qatar Airways Sponsorship	“Visit Saudi” sponsorship globally	Etihad Airways, Emirates Sports Sponsorships
	Within sport	Economic resources (money)	New federation; tournaments	State foundations	Premier Padel	Saudi Arabia Football Federation (SAFF); LIV Golf	Sports' governing federations
	For sport	Economic resources (money)	Own clubs and sport institutions	Sovereign investment funds	QSI (Qatar Sports Investments) owning clubs and competitions (PSG, SCBraga, World Padel Tour)	Saudi Arabia’s Public Investment Fund (PIF), ownership of clubs like Newcastle United and several other Saudi clubs	Abu Dhabi United Group for Development and Investment (ADUG), City Football Group
Politics from sports	Sport for politics	Sport government institutions	Political statements.	Sport institutions, governments	Russia Ban from 2022 Football World Cup		UAE denying Shahar Peer a VISA to compete
	Political Activism in Sport	Athletes, clubs	Political statements	Athletes	Athlete boycotts to 2022 Football World Cup, raising awareness to human rights and talking about Qatar working conditions	Toni Kroos’ criticism of athletes moving to Saudi Arabia	

This new systematization of practice and actors makes it possible to analyze and describe current programs for the political use of sport in greater depth and with more criteria, avoiding the use of ideological and limiting concepts such as sportswashing. At the same time, the new framework is used to discuss and plan new programs. By understanding what is being done and what is being used, it is also possible to analyze what others are doing and what is not being done or used. Finally, each of the practices has particular actors and different levels of legitimization. In that sense, the new systematization also opens a window of opportunity to understand how, and under what incentives, sport actors (governing bodies and athletes) are permeable to its political use and what are the areas of risk and sport values that can be jeopardized due to the new ecosystem.

### 3.1 Diplomacy and politics

#### 3.1.1 Through sport

It refers to the use of sports and sporting events to achieve diplomatic goals, promote international cooperation, enhance a nation's image, or pursue economic development. It involves the strategic leveraging of sports as an effective soft power tool. In other words, it means that the instrument used to achieve a political or economic objective is sport itself or a person from the sport world. It is about doing diplomacy through sport. Its goals can range from improving bilateral relations to addressing global issues such as human rights, peacebuilding, development, and public health.

An historical example is Ping Pong Diplomacy, developed in 1971 during the Cold War. The exchange of table tennis players between the United States and China marked a thaw in their relations, leading to diplomatic talks and the eventual normalization of relations between the two nations.

Gulf countries have also been engaged in this type of diplomatic activities. Qatar, for example, hosted the Peace and Sport cup in 2011. Referred to as “Ping Pong Diplomacy 2.0” by the former president of the International Table Tennis Federation, Adam Sharara, it was an event where ten countries were invited to Doha to compete in an environment of peace, prioritizing peace and sports above political divides while representing a big step for endorsing peace through sports^4^.

Likewise, Saudi Arabia and Iran, nations historically embroiled in proxy conflicts, have initiated a series of peaceful exchanges through soccer. Various gestures of goodwill and mutual respect by Saudi authorities towards the Iranian national team, such as presenting flowers upon arrival at the airport and celebrating their success in the AFC Champions League with a cake at the hotel, have been interpreted as a conciliatory message, fostering reconciliation between the two countries^5^.

In recent years, sports have gained legitimation and prestige as a tool for promoting education, integration, equality, and other diverse social goals. In the United Nations' 2030 Agenda for Sustainable Development, sport appears as a key sector to achieve the goals of the program. On the other hand, the EU Work Plan for Sport 2017–2020 (56] promotes sport as a means to contrast unemployment and other social scourges (19). In this framework, several initiatives and institutions have set up a sport for development and peace (SDP) movement. Organizations as diverse as Monaco's based Peace and Sport^6^ or Qatar's based Save the Dream^7^ are using sport to promote social innovation, improve socio-economic condition and conflict resolutions in conflict-ridden regions.

#### 3.1.2 Within sport

In the same way that sport is an effective means to promote political and social solutions, it is also a stage to popularize and massify political messages. In this sense, there is a diplomacy within sport, where governments use sport events to send political statements or fight political struggles. In this case, sport is not the mean, but the battlefield.

An example of this phenomenon are the boycotts of the Olympic Games during the Cold War. While 67 countries boycotted the 1980 Summer Olympics in Moscow (57) to protest against the soviet occupation of Afghanistan, 18 countries of the eastern bloc boycotted the 1984 Summer Olympic in Los Angeles and organized another competition called Friendship Games (58).

Boycotts remain a diplomatic weapon today and gulf countries are on the spotlight. Prior to the 2022 FIFA World Cup in Qatar, various nations started a boycott campaign aimed at highlighting their apprehensions and objections regarding the labor and environmental standards within the hosting nation. Fans raised banners in stadiums, several influent athletes publicly refused to go to Qatar and members of government criticized this World Cup. However, none of the nations that joined the protest banned their athletes from competing^8^.

Concerning the 2022 FIFA World Cup in Qatar, that several western countries and national federation criticized as mentioned previously, there were no teams that refused to participate, countries that banned the broadcasting of its matches, nor critics taking radical measures.

These policy initiatives demonstrate the limitations and conceptual errors behind the concept of sportswashing. As Soyland and Moriconi (3) point out, the successful political use of sport and, in this case, the winning of the bid to host the World Cup, rather than making it possible to hide domestic political problems leads to greater international exposure, larger external scrutiny and increase criticism from those countries that may be threatened or harmed.

On the other hand, the legitimacy of an alleged sportswasher does not depend on itself. A country might want to organize an Olympic Game to clean its image, but the IOC must choose it as the host. Moreover, a successful sport event depends on the participation of the competitors and, in the case of the Winter Olympic in China, the participant countries, including those who imposed a diplomatic boycott, did not hesitate to send their athletes to participate in the competition.

On the contrary, on November 21, 1973, the USSR refused to play its match against Chile for qualification for the 1974 FIFA World Cup in Germany^9^. The match ought to be played at the National Stadium in Santiago, a venue that the recent dictatorship of Augusto Pinochet had used as a center for detention and tortures. Russia did not show up to play, and Chile, whose team took the field alone and scored a goal in the absence of the rival, qualified for the World Cup.

#### 3.1.3 For sport

There are cases in which sport is not the means, but rather the objective of a political action. To achieve the sporting objective (as in an objective strictly related to sport), governments make their political and diplomatic machinery available. So, sport can be conceived as a tool for the development and promotion of the country, in the form of event organization, or as propaganda in the case of political triumphs.

These objectives can vary from obtaining the venue for a sporting event (such as a Formula 1 race, or the Olympic Games), to massively nationalizing foreign athletes to form a competitive national team, as happened in the case of Qatar. Logically, this can include negative practices, such as the implementation of a state-sponsored and controlled doping program.

Considering that these different types of practices are not watertight and complement each other, the political machinery implemented can include sports celebrities, other public diplomatic actors, and even the participation of sports federations. In the case of applications to host a soccer World Cup, for example, the different candidates define a group of ambassadors that include people from sports.

The sport strategies for development and modernization of Arab countries, that have been questioned as sportswashing, include the use of diplomacy and politics for sport. Two of their pillars are hosting international sporting events and building state-of-the-art sporting facilities and promoting and developing sporting success at the elite level (9). Qatar, Saudi Arabia, and the United Arab Emirates are three clear examples of countries that have been seeking to host an international sporting event and build a strong sport and social infrastructure to modernize the country.

According to the Qatar Olympic Committee [(59): 4], “hosting the 2022 FIFA World Cup will accelerate the development of the objectives of QNV 2030, which aim at transforming Qatar into an advanced country by 2030”. To host the event and receive millions of visitors, Qatar built a new metro system in Doha, roads, a new airport, hospitals, hotels, an entirely new city named Lusail, and seven new state-of-the-art stadiums which were used during the World Cup (60).

On the other hand, Saudi Arabia is building a futurist megacity in the desert called Neom. The city will have a year-round winter sport complex where the 2029 Asian Winter Games will take place (61). Following the same path, the UAE is emerging as a prominent global center for the hosting of significant international sporting events, alongside with the construction of more than 16 football stadiums and “Zayed Sports City”, a world-class sports stadium and events venue^10^.

These countries have also been pushing to become a central part of the global circuit of mega sporting events, including Formula 1 and Moto GP Championship, international Tennis tournaments, Golf masters, cycling tours, equestrian competitions, or the IAAF Diamond League (1, 2, 9).

Sports victories are a source of national pride and a demonstration of strength (62). In this sense, the promotion and development of sporting success has also been important for these countries. One of Qatar's goals has been to improve the performance of Qatari athletes at all levels (3). The country founded the Aspire Academy in 2004 in order to identify and develop talented athletes. The academy has state-of-the art facilities, leading expertise in sports science, as well as highly qualified international scouts and coaches who seek to improve the level of elite Qatari Athletes (63).

Pursuing the same sporting success, Qatar has as well gathered attention for its practice of integrating foreign-born athletes into its national teams [the Qatari national team that won the silver medal in the 2015 Men's Handball World Cup was made up with only two players (out of 16) that were born in the country]. Many of these athletes are provided with the so-called “mission-passports,” temporary documentation subject to a specified legal timeframe, enabling them to represent Qatar while retaining citizenship of their own country^11^.

Nevertheless, Qatar is not the only country from the gulf region concerned about sporting success. Saudi Arabia pursues the same objective, pursuing a place among the 30 best teams in the world in 2030 (1). While this kind of controversy led the IOC and FIFA to amend their regulations on the nationalization of athletes, the problem persists.

### 3.2 Commercial and corporative diplomacy

#### 3.2.1 Through sport

Programs for the political use of sport (or, rather, for social and economic development based on sport) include a series of dimensions that, rather than being related to sports diplomacy, are a clear example of commercial and corporative diplomacy. Commercial and corporative diplomacy is a version of economic diplomacy (64, 65). It is promoted through state measures that lead business leaders and national producers to transcend the country's borders and invest heavily abroad. In this case, on issues directly related to sport.

In this case, sport is used as a showcase or screen to demonstrate the economic power of a country. Economic power is the element used to engage with external actors and achieve the objectives pursued by the country. If the money is missing or is not enough, these goals cannot be met. The key actors are flagship companies, state foundations, or sovereign investment funds that invest in global sport and sponsor sport institutions and events. These actions are supported by their national governments that generate measures and provide support to commercial actions. In some cases, these are entities that receive direct financial support from states.

In this sense, there is a commercial diplomacy through sport, in which flagship companies and organizations use clubs and sport events to promote themselves. Qatar Airways, Qatar Foundation and Qatar Tourism Authority (both from Qatar), Emirates and Etihad Airways (from UAE), NEOM and Aramco (from Saudi Arabia), are examples of companies that have been displayed in top clubs' jerseys or key sport events (1, 2, 9).

In a similar approach, Lionel Messi, one of the best and well-known athletes in the planet, became Saudi Arabia's tourist ambassador and promoter in May 2022, signing a deal of approximately $2 million^12^. This agreement comes from a perspective of Saudi Arabia's government's mission to make the country attractive for travelers all over the world, alongside the movement “Visit Saudi” being widespread globally. Nevertheless, it can also be regarded as part of a strategy to “mask” morally reprehensible situations that take place in the country, such as the labor regime. With many narratives in recent years that made the world conscious about several injustices enacted by the Saudi regime, paying millions and millions for athletes that influence the world to endorse the country can be considered an element of the country's commercial and corporative diplomatic strategy.

#### 3.2.2 Within sport

Economic diplomacy is also practiced within sports, as states create new circuits and competitions outside the normal institutional sphere, with the investments from the Private Investment Fund (PIF) tour as one of the primary showcases on the sphere of gulf states. The PIF, one of the world's largest sovereign wealth funds and a crucial element of Saudi Vision 2030^13^, invests not only in football but also in several other sports, with golf being one of them. The PGA Tour had been the main competition in the golf circuit since 1929 but an opposing one named LIV golf was created in 2021 (17).

The quandary and critic against LIV golf is that it consists, one more time, of stratospheric signing fees and prize pools (including the largest prize ever in the history of golf), convincing golfers to choose to participate in LIV golf instead of the PGA tour (66). Once again not for the competitiveness, but for financial reasons. The administration of LIV Golf claims that the main objectives of the competition are to “improve the health of professional golf” and “unlock the sport's potential” (67), while critics accuse the competition of sportswashing and Saudi Arabia of “buying legitimacy and polish its global image (through money)” (17).

At the same time, Qatar had a similar approach in Padel. With heavy investment from the Qatar Sports Investments (QSI) fund, Qatar converted itself in an important actor in the world of padel by creating an alternative circuit called Premier Padel. This competition is meant to oppose, similarly to LIV golf, the main world competition in Padel, World Padel Tour (WPT)^14^. This created a conflict between the WPT and the International Padel Federation and was a target of heavy criticism due to the unfair competition it generated^15^, as players were persuaded to participate in Premier Padel Qatar due to the high amounts of capital around it.

Private investment funds are impacting the world of sports in ways that were never seen before and are jeopardizing the essential values of sport to transform it into a vehicle for legitimacy strategies of countries that have a morally questionable reputation. “Breakaway leagues” such as LIV Golf, Premier Padel and others are becoming more widespread and frequent and are far from being consensual. Whether by recruiting top athletes through financial persuasion or challenging older and established competitions with sums of investment that they can't compete with, economic diplomacy within sport is threatening the future of sport as we know it.

Nevertheless, investments in sports do not end here. The Qatari government has also founded non-governmental organization whose work is based on sport integrity and the promotion of initiatives on sport as a vehicle for development (8), such Doha's based International Centre for Sport Security (ICSS), established in 2010. The Qatari government and the ICSS also led the efforts to create the Sport Integrity Global Alliance (SIGA) which is the first independent and self-financed international organization dedicated to sports integrity (68)^16^.

#### 3.2.3 For sport

The financial practice in sport goes beyond marketing actions. Due to the current ecosystem of sport hyper commodification (69) that has intensified market understanding and attitudes toward sport, wealthy countries such as Persian Guld autocracies have created branches national sovereign wealth funds to directly invest in sport.

The Abu Dhabi United Group for Development and Investment (ADUG), for instance, purchased Manchester City in 2008 and in 2013 became a global multi-club organization with the creation of the City Football Group (CFG). It currently holds 13 clubs^17^. The Emirates also own the road bicycle UAE Team Emirates^18^, which competes at the UCI World Team level.

Qatar Sports Investments (QSI) became globally well-known since it acquired French football club Paris St-Germain (PSG) in 2011 and took the club to an elite level, winning 9 national titles with stars such as Neymar Jr, Kylian Mbappe or Lionel Messi.

Saudi Arabia was not left behind in this process and, similarly to its neighbors, it managed its Public Investment Fund (PIF) to heavily invest in sport. PIF took over British Premier League club Newcastle United FC and aided in the transfers of a long list of well-known elite football players to transform the Arabian Football League in one of the richest (in terms of payments) in the world.

Furthermore, it is possible to see how Arab countries are also highly influencing and transforming the character of transfer markets within sports (mostly in football) by signing players for record fees and regimenting market values, as a lot of players choose not to compete in better leagues quality-wise due to the wish of earning more money, even if it jeopardizes their professional success. Joining Cristiano Ronaldo, who was the first European player to pursuit this path to Saudi Arabia, a long list of top-quality players joined him in the Gulf. This list includes impactful names in the world of football such as Ballon D'or winner Karim Benzema, N'Golo Kanté, Roberto Firmino, Riyad Mahrez, among many others.

### 3.3 Politics from sport

#### 3.3.1 Sports for politics

Sport is also used to send political messages or take a political position in a conflict. In this case, sport's own governance bodies or athletes take a political measure or perform actions that impact the development and undertaking of sport competitions.

Historically, one of the strongest political statements through sport was the embargo of South Africa throughout the Apartheid times. Due to the system of segregation and discrimination that was in place in the country at the time, the South African heads of state decided to prohibit national sports teams that included white and black athletes to participate in competitions in foreign territory (70), which hindered dozens of South Africans of participating in the next Olympic Games. This segregation led to the action of international sport federations all over the world, who began to impose sanctions and campaigns against South Africa.

In 2022, just one year ago, we witnessed a comparable political situation where sport was used to set up a political statement. Following its invasion of Ukraine, Russia stood in a place of heavy criticism towards the state's political actions, and several measures were undertaken by other states all around the globe with the goal of demonstrating their dissatisfaction with how Russia was acting. The sports world was not immune to this, as many sports organizations and countries decided to send a political message to the world by banning Russia and Russian athletes from participating and competing in certain competitions and sporting events, such as the 2022 World Cup in Qatar, a competition from which Russia was excluded by FIFA after having qualified for the playoffs^19^ (71, 72).

This category can be defined as the use of sport as a platform for launching or amplifying political messages or even political careers. Cases abound of politicians who launched their careers from the world of sport, after succeeding as leaders. For example, the former president of Argentina, Mauricio Macri, after becoming president of Boca Juniors, or the case of Miguel Cartes in Paraguay. There are also cases of sportsmen and women who, after a successful career, use their social capital as a tool to start successful political careers, as in the case of George Weah, among others.

#### 3.3.2 Political activism in sport

The 2022 World Cup hosted by Qatar also led political activism to become louder in sports institutions and athletes to undertake actions on this line. On one side, world-known athletes such as Leon Goretzka, Toni Kroos, Philipp Lahm or Christian Eriksen publicly expressed their displeasure of the decision of such an important tournament for sports being hosted in Qatar^20^.

Conversely, national teams proactively engaged in advocacy efforts. For instance, the Netherlands national team athletes convened meetings with migrant workers to shed light on their poor working conditions. Additionally, North American players displayed a rainbow variant of their national badge during training sessions, symbolizing a stance against Qatar's discriminatory policies towards the LGBTQ community^21^.

Across the globe, during the NBA 2020–21 season, the league and players association agreed to display social justice messages related to the BLM movement on the back of players' jerseys. Messages such as “I can't breathe”, “Power to the people”, “See us”, “Speak up”, “Justice”, “Peace”, among others, intended to call the world's attention to systemic racism and police brutality^22^. Similarly, in the NFL, players were allowed to present slogans on their helmets that portrayed political activism, such as “End Racism” or “Stop Hate”, and kneeing during the national anthem became a usual practice^23^. These efforts demonstrated how sports leagues and their athletes also highlight political messages to protest, achieve global awareness and promote activism.

## 4 Discussion

This paper systematizes the current political uses of sport. The results show that what has often been grouped under the umbrella concept of sport diplomacy is, actually, a set of varied practices with different means and objectives. This novel framework for analysis has a twofold practical utility. First, it serves to analyze, in a broader and deeper way, the political programs that use sport as a soft power tool. There are practices in which sport is an effective tool for achieving social, political, and economic objectives. In short, sport is understood as a type of soft power. In other occasions, the sport world is the target of a political action. To achieve sport success, to host sport events, or even to have a national citizen in front of an international sport federation might generate and pave the way for obtaining social goals as diverse as promoting national culture, generate economic development, or gain political influence in the international order. In this sense, the systematization provided by this article also serves as a roadmap for the political planning of successful strategies that use sport as a diplomatic and commercial tool.

The article also evidences that concepts such as sport diplomacy or sportswashing still lack a wider theorization and a better operationalization. More than talking about a sport diplomacy in singular, it might be better to distinguish between sport diplomacy through, for and within sports, in order to understand sports diplomacies in plural.

There are occasions where money becomes the drive for political programs related with sport issues. In this case, money is the soft power tool. In those cases, where economic resources become the lubricant of political action, it is more correct to speak about economic or commercial diplomacy.

Many of the actions by which wealthy autocracies are accused of sportswashing are promoted and based on the use of money as the means to achieve their goals. In some cases, economic diplomacy goes even beyond what could or should be achieved through sports diplomacy. A clear example is obtaining the rights to organize a sporting event or the television broadcasting of an event by bribing key institutional players. The key factor to achieve these goals is money and not diplomacy.

In this sense, this article provides information and evidence on two key questions related to the debates about current spurious political use of sport. First, it reveals that the concept of sportswashing is inadequate. The successful political utilization of sport, rather than masking domestic political situations, significantly increases external scrutiny. So, it brings the world's eyes into domestic political issues such as labor market regulations, gender politics, or human right protections.

Secondly, it exposes that there is no possibility for the unmediated and spurious use of sport in a one-sided manner. Tolerance and legitimacy from other parties, mainly sport governance bodies (which always have the final word), are always required. The supplanting of public diplomacy by economic diplomacy to obtain rights to sporting events would not be possible if there were no corruptible, bribe-seeking actors in the sporting world. As Moriconi (9) explains, what is usually questioned as sportswashing are political practices and actions that, in order to be effective, always require, at some point, the tolerance and legitimization of audiences, supporters, in-field sport actors, management, official and sport governance bodies. It is impossible for a country to continue to host a Formula 1 Grand Prix if the FIA does not agree, for whatever reason, to include this race in the championship. A football championship, no matter how many stars it has, can hardly survive if the public and sponsors turn their backs on it.

The history of the political use of sport itself, as this article demonstrates, is full of possibilities for boycotting or opposing the spurious use of sport or actions that could jeopardize the essential values of sport. Bayern Munich fans, for instance, opposed the sponsorship agreement with Qatar Airways because they felt that the club's values did not correspond with the Gulf country's domestic political controversies.

What are the real values that drive the sports industry and sport management? What are supporters and audiences willing to tolerate and legitimate?

In this sense, this article contributes to the current debate on the path that sport governance is taking, in a world marked by sport hyper commodification, and the key place that sport has taken in the development programs of governments, far removed from democratic values.

The prestige of sport as a tool for social intervention and the promotion of good values is not related to the very essence of sport, but to the social and political use made of it. If the political use of sport is motivated by negative values, there is nothing to prevent this prestige from disappearing. In any case, this will not depend only on one actor, whether it is autocratic or democratic, but on the acceptance and tolerance of most of the sport ecosystem.

## Footnotes

^1^See https://basketnews.com/news-199924-motiejunas-evaluates-dubais-euroleague-entry-for-next-season-serbian-clubs-future.html

^2^See https://www.theguardian.com/world/2023/sep/21/mohammed-bin-salman-says-he-will-continue-doing-sport-washing-for-saudi-arabia

^3^ https://www.fifa.com/fifaplus/en/member-associations/france/articles/world-cup-qatar-2022-greater-than-stephanie-frappart-in-history-as-first-female-referee-in-costa-rica-v-germany

^4^ https://www.infobae.com/aroundtherings/federations/2021/07/12/president-welcomes-ping-pong-diplomacy-20/

^5^ https://gulfif.org/can-soccer-diplomacy-mend-fences-between-iran-and-saudi-arabia/

^6^ https://www.peace-sport.org/

^7^ http://savethedream.org/

^8^ https://www.euronews.com/2022/11/10/fifa-and-qatar-rattled-as-european-world-cup-boycott-gathers-pace

^9^ https://theathletic.com/5073149/2023/11/21/chile-ussr-fifa-pinochet-50/

^10^ https://www.gulftoday.ae/sport/2021/05/12/uae-a-global-hub-for-hosting-and-organising-major-international-sporting-events

^11^https://merip.org/2022/11/constructing-qatari-citizenship-in-the-shadow-of-the-world-cup/.

^12^ https://www.nytimes.com/2023/06/18/sports/soccer/lionel-messi-saudi-arabia.html

^13^ https://www.pif.gov.sa/

^14^ https://www.elconfidencial.com/deportes/padel/2022-03-14/al-khelaifi-qatar-world-padel-tour-damm-paris-saint-germain_3390787/

^15^ https://www.elconfidencial.com/deportes/padel/2022-05-28/world-padel-tour-demanda-federacion-qatar-sports_3432546/

^16^ https://siga-sport.com/

^17^ https://www.cityfootballgroup.com/

^18^ http://www.uaeteamemirates.com/

^19^https://sportstar.thehindu.com/football/fifa-world-cup/news/qatar-2022-why-is-russia-not-playing-fifa-world-cup-ukraine-invasion-playoff-uefa-ban/article66040225.ece.

^20^https://www.si.com/soccer/2022/11/15/world-cup-qatar-controversies.

^21^https://www.theguardian.com/football/2022/nov/14/usmnt-rainbow-logo-qatar-world-cup-2022-soccer-lgbtq.

^22^https://www.nba.com/news/nba-union-approve-social-justice-messages-jerseys.

^23^https://www.nytimes.com/2021/09/05/sports/nfl-social-justice.html.
